# Are the children alright? A systematic review of psychological adjustment of children conceived by assisted reproductive technologies

**DOI:** 10.1007/s00787-022-02129-w

**Published:** 2022-12-29

**Authors:** Francis Anne Teplitzky Carneiro, Valéria Leong, Sara Nóbrega, Fernando Salinas-Quiroz, Pedro Alexandre Costa, Isabel Leal

**Affiliations:** 1 William James Center for Research, ISPA–University Institute, 1100-304 Lisbon, Portugal; 2 ISPA–University Institute, 1100-304 Lisbon, Portugal; 3 Faculty of Social and Behavioural Sciences, Leiden University, 2311 EZ Leiden, The Netherlands; 4 Eliot-Pearson Department of Child Study and Human Development (EPCSHD), School of Art and Sciences, Tufts University, 02155 Medford, USA

**Keywords:** Childhood, Reproductive donation, IVF/ICSI, Well-being

## Abstract

The present systematic review aims to assess the psychological adjustment of children born through assisted reproductive technologies (ARTs) and to screen for clinical problems when compared with normative data from the standardized indexes of mental health. Following PRISMA guidelines, the search was conducted from inception through September 2021 using APA PsycInfo, APA PsycArticles, Psychology and Behavioural Sciences Collection, Academic Search Complete, Pubmed, Scopus, Web of Science, Scielo, and RCAAP. Search terms related to ART and children’s psychological adjustment were combined to Boolean operators to identify relevant published studies in English, French, Italian, Portuguese and Spanish. Peer-reviewed studies focused on the psychological adjustment of ART children aged between the 3 and 11 years were included. From a total of 337 results, 45 papers were eligible to be included in this review. Data extraction was performed independently by two authors and revised and confirmed by other two authors. All children scored below the clinical range for psychiatric symptoms when compared with normative data for the Strengths and Difficulties Questionnaire (SDQ) or the Achenbach System of Empirically Based Assessment (ASEBA), regardless of type of ART and different family configurations. Further, some evidence suggests that surrogacy children with gay fathers present the lowest levels of psychological problems when compared to normative data. These findings enable practitioners to develop an informed view of ART children mental health outcomes to help parents find more adaptive strategies to navigate their chosen pathways in healthier ways.

## Introduction

The use of assisted reproductive technologies (ART) has been increasing dramatically over the last 40 years, due to advancements in science and technology, fertility decreases in young women, delayed childbearing, shifts in societal attitudes, changes in legislation, among others [1, 2]. It is estimated that since the birth of the first “test-tube” baby in 1978, over 8 million children have been born through ART [3]. According to the European Society of Human Reproduction and Embryology, 157,500 children were born via ART in 2015 [4]. These procedures can be described as a wide range of medical techniques that are used to, primarily, help infertile couples conceive. A variety of ART treatments are available. In some of these procedures, children have both a gestational and a genetic connection to their mother, father and the mother that undergoes the pregnancy. This is the case of homologous in vitro fertilization (IVF; fertilization of an egg and sperm in a Petri dish) and homologous intracytoplasmic sperm injection (ICSI; the injection of a single sperm directly into a mature egg). For other procedures—included in the reproduction donation—children can be genetically related only to the mother, if a donor sperm is used (donor insemination; DI); only to the father via egg donation (ED); or to neither parent when both the donor egg and sperm are used) (embryo donation) [5]. Parents may also choose surrogacy, in which the pregnancy is carried by another woman. In this case, depending on the specific arrangement, children can either be genetically related to only one parent, both parents, or neither [5]. The various possibilities allowed by the available techniques of reproductive donation involve different genetic relatedness to mother and/or father, which may imply dissimilar effects on children’s psychological adjustment. The majority of the studies reported finding no significant differences in children’s psychological adjustment between different ART children; nevertheless, the different particularities and family processes associated with the absence of genetic relatedness to mother and/or father and its impact on children’s psychological adjustment remain unexplored.

Moreover, reproduction donation is becoming an increasingly popular method for conceiving children not only by infertile couples, but also by same-gender couples or single parents [6]. For this reason, by the end of the twenty-first century, new family forms began to appear. The term “new families”, proposed by Golombok [7], distinguishes these from other “non-traditional families”, such as those formed through cohabitation or single parenthood, about which there is already a considerable amount of literature [7, 8]. A heterosexual, cisgender couple raising children that are genetically related to them is considered the “traditional family” paradigm. In that sense, new families may present different structures in terms of number, gender, and parents’ sexual orientation, as well as in their genetic and gestational relatedness with the children [1]. Solo mothers, also known as single mothers by choice, are a relatively new type of “non-traditional family” that is formed by single women who actively decide to create a family without having a partner [9]. For example, in 2017 about one-in-five children (21%) in the USA were living with a solo mother [10]. Several studies show that single mothers by choice are usually well-educated women in professional occupations who become mothers in their late 30 s or early 40 s. Many choose to become single mothers not because they do not want a traditional family setting, but due to their increasing age and its associated fertility decline [8].

Despite that lesbian-parented families may differ from the previous family configuration because of the mothers’ sexual orientation, they share the similarity of children being raised by women without the presence of a father. The increase in births among lesbian women in the USA has been so enormous and many designated it as a “lesbian baby boom” [11]. According to a 2001 US survey, 74% of lesbian couples are treated in ART facilities [12]. Operationalizing the concept of parenthood for lesbian-parented (and gay-parented) families is complex, since there are many possibilities for the parental status. For instance, for a lesbian couple with a child conceived through sperm donation, the non-biological mother is recognized as a mother due to the emotional connection, but the lack of biological connection could implicate a lack of legal status regarding the child, depending on the country’s legal context. This ambiguity interferes with the estimates of the prevalence of lesbian-parented families and to access a total description of those families [13]. Nonetheless, it is estimated that, in 2014, in the USA, 157,700 children were being raised by lesbian-parented families, in addition to 150,000 children being raised by a lesbian single parent [14].

In gay-parented families, a more recent “new family” configuration, children are raised by gay couples or single gay parents, although there are fewer studies about gay-parented families because most children in same-gender households are being raised by lesbian mothers. Most of the research on gay-parented families has focused on those formed through surrogacy, in which the parents can select an “open-identity donor” (with whom they might have contact in the future) or an “anonymous donor” (with whom they want little or no contact) [15]. In 2014, in the USA, around 32,300 children were being raised by gay couples, in addition to 60,000 children being raised by a gay single parent [14].

Lastly, trans-parented families are the least known family configurations. Nevertheless, survey data show that between 25 and 49% of trans individuals are parents, even if children may be born before or after the parent’s transition. Trans parents are, generally, older than trans individuals without children and the prevalence of parenthood tends to be higher among trans women than among trans men [16].

In recent decades, there has been a considerable increase in non-traditional families. Therefore, an increasing body of research on the medical outcomes of children born through ART has emerged out of concerns for the well-being of children. Consequently, several studies have started to focus on the psychological adjustment of children born through ART [17]. Psychological adjustment is described as an individual's general sentiments of well-being and pleasure as a result of reduced stress in their daily lives [18]. Further, Ward and Kennedy [19] described psychological adjustment in terms of emotional and affective results, including mental health concerns (e.g., depressive symptoms, temper disturbances, etc.) [19]. Because ART households differ from conventional families, extra concerns regarding children’s psychological adjustment arise, due to the distinct pattern of gestational and genetic connections between people inside and outside the nuclear family. In that sense, planned families through ART are often victims of social criticism and stigma. For example, due to the traditional belief that “a child needs a father”, there is a tendency to suppose that children from solo mothers are not as psychologically adjusted as those in traditional households [20]. Similar preconceived notions affect DI families, especially in cases where the donor’s identity is unknown to the child as they grow up. The secrecy of the child’s biological origins is thought to result in negative psychological consequences for children [21].

Further, concerns regarding gay-parented families are also highly prevalent. This is due to the fact that fathers are rarely the primary caregivers and are often seen as less nurturing and capable of parenting than women [22]. Particularly gay-parented families that use surrogacy are often stigmatized, as many assume that the child may view the surrogate as a mother and suffer from the absence of this relationship, or feel abandoned by her [15]. In contrast to the popular belief, a meta-analysis of the impact of gay fathers on children's psychological adjustment discovered that when compared to children of heterosexual parents’ children of gay fathers might even do better in some psychological sectors, notably displaying fewer internalizing and externalizing problems [23].

Finally, Golombok [24] has recently developed a theoretical review about the psychological adjustment of children born through ART and concluded that gender, number, genetic relatedness, and sexual orientation of parents do not impact children’s psychological adjustment [24]. Instead, similar to those in traditional households, the psychological adjustment of children raised in ART families appear to rely upon the parents’ well-being, the nature and quality of relationships inside the family, the assistance of their wider community and the social occasions they are raised in [24]. Although the authors have included several studies on the field in the last 40 years, it was not systematically conducted. A systematic review was indeed performed by the mentioned author [25]; however, it was a review focused on adolescents and only with studies published in English. Therefore, it is important to systematically summarize the immensity of the data about the psychological adjustment of ART children under 11 years old and to assess the quality of studies. Furthermore, many of the studies in the field have only performed intra-samples comparisons (ART children versus natural conceived children and adoptive children) which could add an important bias to the study’s conclusions. A confirmation that all these studied children are in fact well adjusted, when compared with normative data for the main indexes of mental health is lacking. It is important to perform a mental health screening of the ART children across all studies (when possible) enabling a homogenization of the information regardless of the heterogeneity of the methodologies, samples, sampling methods, countries, and informants.

In sum, to the best of our knowledge, no systematic review has examined the psychological impact that being born through ART has on children, and if ART children’s psychological adjustment could be considered similar to that of NC children.

The general objective of the present study is to systematically assess the psychological adjustment of children aged between 3 and 11 years born through ART. The first specific objective is to compare ART children’s scores on standardized indexes of mental health with normative data to examine ART children’s overall psychological adjustment. The second specific objective is to examine possible differences in children’s psychological adjustment across different ART, and especially those involving reproductive donation (DI, embryo donation, egg donation, sperm donation and surrogacy) where the child is not genetically related to at least one parent. Lastly, the third specific objective is to examine possible differences in children’s psychological adjustment across different family configurations, namely, between children from single mothers/fathers’ families versus children from two-parent families; children from lesbian-parented families and/or gay-parented families versus children from heterosexual-parented families; and children from transgender-parented families versus children from cisgender-parented families. Summarizing the mentioned literature will help to identify what is known about children’s psychological health in ART families, as well as to help identify gaps in the literature for future research in this field.

## Methods

### Search strategy

A systematic review of children’s psychological adjustment in ART families was developed. The systematic search followed PRISMA guidelines [26]. A literature search was conducted independently by two researchers (V. L. and S. N.) using the APA PsycInfo, APA PsycArticles, Psychology and Behavioral Sciences Collection, Academic Search Complete, Pubmed^®^ (National Library of Medicine), Scopus^®^ (Elsevier), Web of Science version 5.35 (Clarivate Analytics), Scielo (Scientific Electronic Library Online) and RCAAP (Repositórios Cientificos de Acesso Aberto de Portugal) (see Table 1). Search terms are described in Table 1. Search terms also incorporated some MeSH terms and comprised all possible key words related to ART and children’s psychological adjustment. Additional studies were identified through the lists of references of the included studies and through references from field expert’s curriculum.

**Table 1:** Search and study selection for a systematic review on children’s psychological adjustment in ART families

Search key words (all in title/abstract and key words)	Eligibility
Exposure: [Assisted reproduct* OR in vitro fertilization OR in vitro fertilisation OR IVF OR sperm don* OR egg don* OR semen don* OR insemination OR gamete don* OR embryo don* OR ICSI OR intra-cytoplasmic OR Surroga* OR planned families OR LGBT OR gay OR lesbian] AND Outcome: [(child*) AND (psycho* adjustment OR adjustment OR well-being); Exposure: [Procreazione artificiale OR procreazione assistita OR fecondazione in vitro OR FIV OR dona* sperma OR dona* semen OR Inseminazione OR dona* ovuli OR dona* gameti OR dona* embrioni OR ICSI OR intracitoplasmatica OR Surrogata OR familiari pianificata OR LGBT Or Gay OR Lesbica] AND Outcome: [(Bambino) AND (Adattamento psicologico OR Adattamento OR Benessere)]; Exposure: [Reprodu* Asistida OR Fertilización in vitro OR FIV OR Dador de esperma OR Dador de óvulos OR Dador de semen OR Inseminación OR Dador de gametas OR Dados de embrión OR ICSI OR Intracitoplasmática OR Maternidad por sustitución OR Famílias planeadas OR LGBT OR Gay OR Lesbiana] AND Outcome: [(Niñ*) AND (Ajuste psicológico OR Ajuste OR bienestar)]; Exposure: [Reprodu* assistida OR fertilização in vitro OR FIV OR dador esperma OR dador de óvulos OR dador óocitos OR dador de sémen OR Inseminação OR dador gametas OR dador embri* OR ICSI OR intracitoplasmática OR maternidade por substituição OR barriga de aluguer OR famílias planeadas OR LGBT OR gay OR lésbica] AND Outcome: [(criança*) AND (ajustamento psicológico OR ajustamento OR bem-estar)]	Inclusion criteria: a) Empirical articles with available abstract published in peer-review journals b) Papers published in English, French, Italian, Spanish and Portuguese and in peer-reviewed journals c) Quantitative and mixed-methods studies d) Studies focused on ARTs as defined in the search keywords e) Studies focused on children aged between 3 and 11 years old. We decided to include both early childhood (3- 6 years) and middle childhood (6–11) as defined by Papalia and colleagues (2008) f) Studies focused on children psychological adjustment Exclusion criteria: a) Review articles b) Qualitative studies c) Papers that do not focus on children (3–11) d) Non-medical procedures (e.g., self-insemination)

### Study selection

Gamete/embryo donation and surrogacy are relatively recent practices—as mentioned by Ilioi and Golombok [7]—and to our knowledge, no previous review has examined the psychological outcomes of children from ART; therefore, we decided not to impose any limits to time of publication. Thus, the literature search was conducted from inception through September 2021. Inclusion and exclusion criteria are described in Table 1. Articles that used the following terms for psychological adjustment were also included: child development, emotional and/or behavioural adjustment, socioemotional development, child adjustment, psychological development, and well-being. ART was defined as IVF, ICSI, donor insemination, gamete donation, egg/oocyte donation, sperm donation, embryo donation and surrogacy. This review was not limited to studies published in English, but has also searched for studies published in four other languages (Portuguese, Italian, French and Spanish) and was focused on children aged between 3 and 11 years not only to expand upon [25] systematic review that focused on the psychological adjustment of adolescents born through ART (11–18 years old), but also to focus throughout all childhood, which includes the early childhood (3–6 years) and middle childhood (6–11) as defined by [27].

### Screening, quality assessment, data extraction and data synthesis

**Fig. 1:**
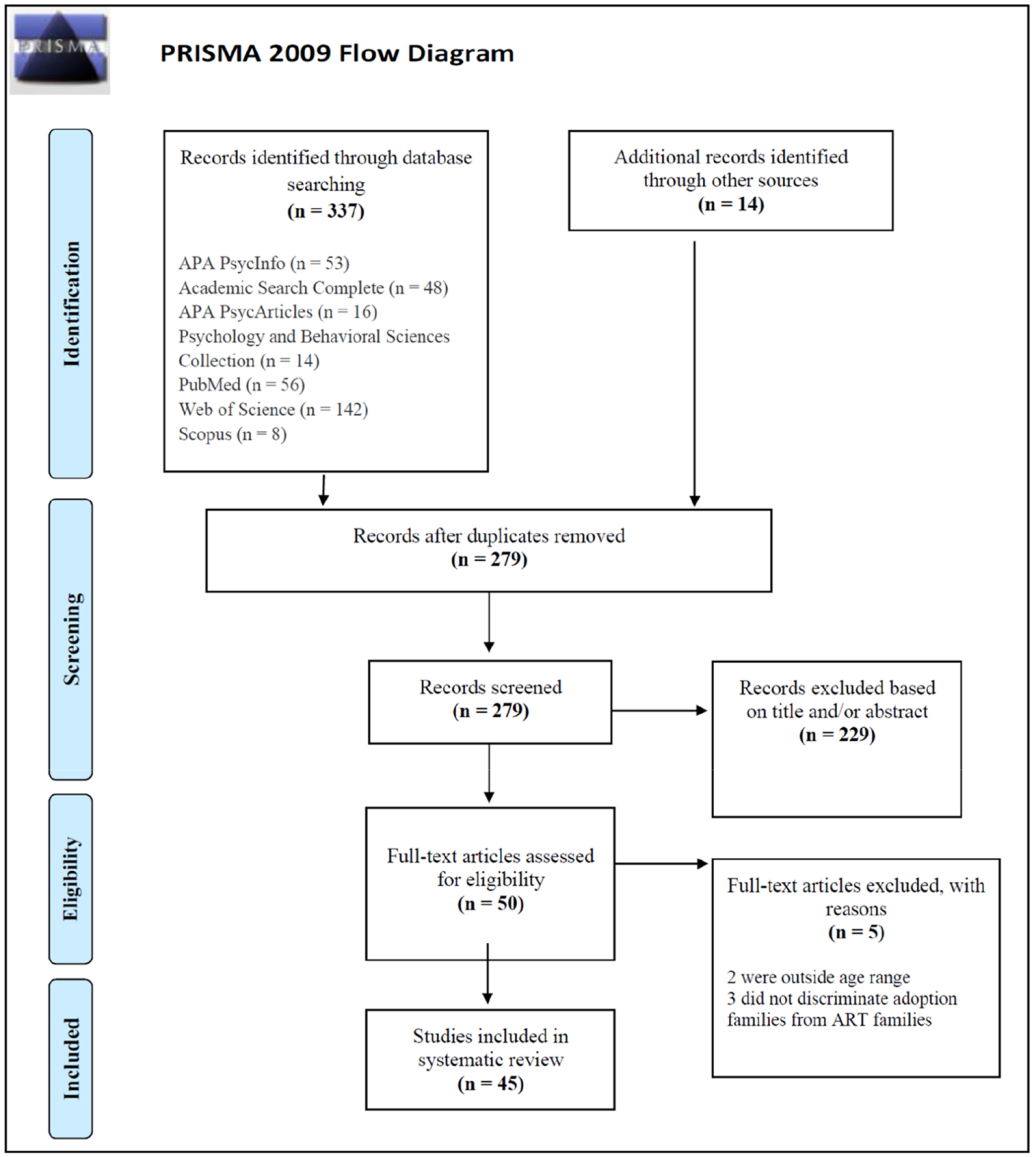
PRISMA flow diagram for the systematic review of children’s psychological adjustment in ART families

As shown in Fig. 1, a total of 337 results were obtained. Two independent authors (V.L. and S.N.) performed an initial inspection of the titles and abstracts of each study. Duplicates (*n* = 44) were removed and additional studies from other sources (*n* = 14) were included. The 279 studies were then assessed in accordance with the inclusion and exclusion criteria. From an initial screening, 229 studies were excluded based on the title and/or the abstract. This process was repeated by the two mentioned authors to confirm the first inspection. The remaining 50 studies were thorough analysed independently by three authors (F.A.C., V.L. and S.N.) and after applying the exclusion criteria five studies were excluded. Lastly, the authors independently revised the remaining articles to confirm their eligibility and performed a formal quality assessment (see *Appendix A* available in the OSF, at https://osf.io/pjnbk/?view_only=92979f986dfc419297eed6a8847cda1e). The quality assessment final table was developed by the authors based on available scientific criteria to assess empirical studies' quality and was also based on some criteria of the Critical Appraisal Skills Programme standards [28]. Since there are no CASP criteria specifically for quality assessment of quantitative studies, we used some criteria of qualitative studies and adapted to both qualitative and quantitative studies to evaluate all included articles. Divergences were discussed and resolved by consensus among three authors (F.A.C., V.L. and S.N.). The present systematic review included 45 studies.

The publication year, descriptive data details (country, ART type, family configuration, children’s age, measures, psychological adjustment definitions), study design and main outcomes were extracted and summarized. All measures extracted for each study were organized by informant (parents, teachers and/or children). Regarding the measures of psychological adjustment, the vast majority of the studies used the Strengths and Difficulties Questionnaire (SDQ) and ASEBA forms (CBCL and TRF) and the few other measures extracted were the Rutter scales, the Pictorial Scale of Perceived Competence, the Eyberg Child Behaviour Inventory (ECBI), the Rosenberg Self-esteem Scale and the Social Skills Rating System (SSRS). The mean scores (and standard deviation) of the SDQ and ASEBA forms (CBCL and TRF) were also extracted (see Appendix B available in the OSF, at https://osf.io/pjnbk/?view_only=92979f986dfc419297eed6a8847cda1e). Specifically, the scores of the Internalizing (Emotional Problems and Peer scales), Externalizing conduct and Hyperactivity/Inattention scales) and total difficulties scales of the SDQ. For the ASEBA forms, the scales were also the Internalizing, Externalizing and Total Behavioural Problems. Data extraction was performed independently by two independent authors (V.L. and S.N.) and later two other authors (F.A.C. and P.A.C) revised and confirmed the extracted information.

Concerning data synthesis, the SDQ and ASEBA mean scores of the included studies were organized to perform a comparative analysis with normative data for all studies, including those that only used comparison samples (e.g. naturally conceived children). Finally, using the program MAXQDA (2020), we organized the data of the main outcomes into the following sections and subsections: (a) ART children and different ART; and (b) ART children and family configurations, which included (b.1) the number of parents and ART children’ psychological adjustment; (b.2) lesbian-, gay- and heterosexual-parented families and psychological adjustment; and (b.3) trans parents and ART children’s psychological adjustment.

## Results

### Descriptive overview of the studies

Table 2 is organized to include the studies by the first author, year of publication and country of sample recruitment. The study design, ART type, family configuration and main outcomes of the mentioned papers are also summarized. In the results section, the included studies will be referenced as SN and the respective study number accordingly to Table 2 (extended version on appendix C available in the OSF, at https://osf.io/pjnbk/?view_only=92979f986dfc419297eed6a8847cda1e).

**Table 2:** Summary of the studies on the psychological adjustment of children born through ART

Study number	Authors, year and country	Study design	ART	Family configuration	Main outcomes
1	(a) Golombok et al. (1996). UK, Italy, Spain and The Netherlands	Mixed methods (qualitative, quantitative); longitudinal/ follow-up	IVF and DI	462 heterosexual coupled parents: 116 IVF, 111 DI, 115 adoptive and 120 NC families	DI Dutch children showed more evidence of emotional/behavioural problems compared with DI children from other countries
2	Brewaeys et al. (1997). The Netherlands	Mixed methods (qualitative, quantitative); cross-sectional	DI	98 families: 30 lesbian DI coupled mothers, 38 heterosexual DI coupled parents and 30 heterosexual NC coupled parents	Children’s emotional/behavioural adjustment in lesbian mother families presented similar scores than a large Dutch population sample Children from heterosexual DI families presented more emotional/behavioural problems
3	(a) Brewaeys et al. (1997). The Netherlands	Mixed methods (qualitative, quantitative); cross-sectional	IVF and DI	98 heterosexual coupled parents: 38 DI, 30 IVF and 30 NC families	DI children presented more emotional/behavioural problems compared to NC and IVF children from study and a large Dutch sample
4	Chan, Raboy & Patterson (1998). USA	Quantitative; cross-sectional	DI	80 DI families: 34 lesbian coupled mothers, 21 lesbian single mothers, 16 heterosexual coupled parents and 9 heterosexual single mothers	ART Children were reported as being well adjusted
5	Montgomery et al. (1999). USA	Quantitative; cross-sectional	IVF	692 families (probably heterosexual coupled parents);	This large group of IVF children presented normal psychological development with no significant behavioural or emotional problems
6	Golombok & Murray (1999). UK	Mixed methods (qualitative, quantitative); cross-sectional	Egg donation, DI and IVF	162 heterosexual coupled parents: 41 IVF, 45 DI; 21 egg donation and 55 adoptive families	The families presented no significant differences related to children's psychological adjustment
7	Hahn & DiPietro (2001). Taiwan	Quantitative; cross-sectional	IVF	113 single mothers: 54 IVF and 59 NC families	Psychological functioning of IVF children was similar to NC children
8	Golombok et al. (2001). UK	Mixed methods (qualitative, quantitative); longitudinal/ follow-up	IVF	121 heterosexual coupled parents: 34 IVF, 49 adoptive and 38 NC	IVF children presented a good functioning and similar emotional adjustment than NC children and adoptive children
9	(a) Golombok et al. (2002). UK, Italy, Spain and The Netherlands	Mixed methods (qualitative, quantitative); prospective longitudinal/ follow-up	IVF and DI	400 heterosexual coupled parents: 102 IVF, 94 DI, 102 adoptive and 102 NC families	ART children were similar to the adoptive and NC children on any of the measures of psychological adjustment
10	Tully et al. (2003). UK	Mixed methods (qualitative and quantitative); cross-sectional	IVF and OI	242 heterosexual coupled parents:121 IVF/OI and 121 NC families	No significant differences between the IVF/OI and NC twins in internalizing, externalizing scores
11	Bos et al. (2004). The Netherlands	Quantitative; cross-sectional	Not specified	100 planned lesbian coupled mothers	ART children presented a good adjustment Biological and social mothers presented no significant differences in their reports about their child’s emotional/behavioural adjustment
12	(b) Lycett et al. (2004). UK	Mixed methods (qualitative, quantitative); longitudinal/ follow-up	DI	46 heterosexual coupled parents	Mothers from disclosing families considered their children to show a lower level of conduct problems No significant group differences for child adjustment
13	Golombok et al. (2006). UK	Mixed methods (qualitative, quantitative); longitudinal/ follow-up	Surrogacy, DI and oocyte donation	183 heterosexual coupled parents: 34 surrogacy, 41 DI, 41 oocyte donation and 67 NC families	There were no significant differences between family types for the children's psychological well-being
14	MacCallum et al. (2007). UK	Mixed methods (qualitative, quantitative); cross-sectional	Embryo donation and IVF	79 heterosexual coupled parents: 21 embryo donation, 30 IVF and 28 adoptive families	No differences were found between embryo donation children and IVF children
15	Bos and Hakvoort (2007). The Netherlands	Quantitative; cross-sectional	DI	100 lesbian coupled mothers: 42 self-insemination with known donor and 58 DI families	Children with a known and an unknown donor presented no differences on internalizing and externalizing behaviours
16	(c) Bos et al. (2007). The Netherlands	Mixed methods (qualitative, quantitative and observational); cross-sectional	Not specified	200 families: 100 lesbian coupled mothers and 100 heterosexual coupled parents	Child adjustment was not related with family type
17	Golombok et al. (2007). France	Mixed methods (qualitative, quantitative); cross-sectional	IVF/ICSI	55 heterosexual coupled parents: 10 families with triplets, 15 families with twins and 30 families with singletons	There were no differences in children's emotional and behavioural problems due to family type
18	(d) Bos et al. (2008). The Netherlands and USA	Mixed methods (qualitative, quantitative); longitudinal/ follow-up	DI and other not specified ART	152 lesbian coupled mothers: 78 USA and 74 Netherlands families	The Dutch children demonstrated fewer emotional and behavioural problems comparatively to the American children
19	(c) Bos & van Balen (2008). The Netherlands	Quantitative and longitudinal/follow-up	Not specified	63 lesbian coupled mothers	Children from lesbian families presented no significant differences on conduct problems, emotional symptoms and hyperactivity when compared with non-clinical children of similar age
20	(d) Bos et al. (2008). USA	Mixed methods (qualitative, quantitative); longitudinal/ follow-up	DI	78 lesbian coupled mothers	The children in planned lesbian families presented no significant differences in psychological adjustment when compared with their age-matched peers
21	Shelton et al. (2009). UK and USA	Quantitative; cross-sectional	IVF; sperm donation, egg donation, embryo donation and surrogacy	769 heterosexual parent families: 386 homologous IVF; 182 IVF with sperm donation;153 IVF with egg donation; 27 IVF with embryo donation; 21 IVF with gestational surrogacy families;	ART Children when compared to NC children, do not differ in their levels of psychological adjustment nor they appear to be at higher risk of psychological adjustment problems in middle childhood
22	Golombok et al. (2011). UK	Mixed methods (qualitative, quantitative, observational); longitudinal/ follow-up	Egg donation and DI	122 heterosexual coupled parents: 32 egg donation, 36 DI and 54 NC families	No differences were found for child adjustment between family types
23	(e) Golombok et al. (2011). UK	Mixed methods (qualitative, quantitative, observational); longitudinal/ follow-up	Egg donation and surrogacy	118 heterosexual coupled parents: 32 surrogacy, 32 egg donation and 54 NC families	Children from different family types with no significant differences on their psychological adjustment
24	Shechnre et al. (2011). Israel	Quantitative; cross-sectional	DI	76 families: 15 lesbian DI single mothers; 21 lesbian DI coupled mothers; 16 heterosexual DI single mothers; 24 two-heterosexual NC coupled mothers	Children from single parents when compared with children from two-parent families presented more externalizing behaviour problems and aggressiveness Children from lesbian families reported higher levels of prosocial behaviours than children from heterosexual families
25	(b) Freeman & Golombok (2012). UK	Mixed methods (qualitative, quantitative); longitudinal/ follow-up	DI	30 heterosexual parent families	DI families appeared to be functioning well at early adolescence with no significant differences between families where the child had been told about their donor origins
26	(e) Golombok et al. (2013). UK	Mixed methods (qualitative, quantitative); longitudinal/ follow-up	Egg donation, surrogacy and DI	149 heterosexual parent families: 30 surrogacy, 31 egg donation, 35 DI and 53 NC families	Children born through egg and sperm donation presented a good psychological adjustment Surrogacy children had higher levels of adjustment difficulties at age 7
27	Anderson et al. (2014). USA	Quantitative; cross-sectional	IUI, IVF and ICSI	198 heterosexual couples; 7 same-sex female couple and 1 single parent	No differences were found on emotional problems between school-aged ART twins and singletons; however, twins presented fewer behavioural and attention problems than singletons
28	Baiocco et al. (2015). Italy	Quantitative; cross-sectional	Surrogacy and DI	80 families: 20 gay surrogacy coupled fathers, 20 lesbian DI coupled mothers and 40 heterosexual NC coupled parents	Children from lesbian and gay families presented similar levels of emotion regulation and psychological well-being, comparatively to children raised by heterosexual families
29	Anderson et al. (2015). USA	Quantitative; cross-sectional	IVF	198 families: 195 heterosexual coupled parents, 2 lesbian coupled mothers and 1 lesbian single mother	ART twins’ and singletons’ psychological adjustment were within normative ranges
30	Tornello et al. (2015). USA	Quantitative; longitudinal/follow-up	Surrogacy and donor arrangements	Gay coupled fathers: 335 fathers (Wave 1) and 176 fathers (Wave 2)	For gay fathers, the discrepancies between actual and household and childcare division of labor were not associated with children’s adjustment
31	Bos et al. (2016). USA	Quantitative; longitudinal/follow-up	Not specified	190 families: 95 lesbian coupled mothers and 95 heterosexual coupled parents	No differences were observed between household types and child outcomes
32	(f) Golombok et al. (2016). UK	Mixed methods (qualitative, quantitative and observational); longitudinal/ follow-up	DI	103 families: 51 heterosexual single mothers and 52 heterosexual coupled parents	There were no differences between family types and child adjustment, suggesting that single motherhood does not result in psychological problems for the children
33	Bos et al. (2018). The Netherlands	Quantitative; cross-sectional	Not specified	190 families: 43 lesbian coupled mothers, 52 gay coupled fathers and 95 heterosexual coupled parents	No significant differences on children’s psychological well-being due to different family configurations
34	Golombok et al. (2018). USA	Mixed methods (qualitative, quantitative and observational); cross-sectional	DI and surrogacy	95 families: 40 gay surrogacy coupled fathers and 55 lesbian DI coupled mothers	ART Children with gay and lesbian parents presented high levels of adjustment and very low internalizing problems comparatively to the cutoff point for clinical problems
35	Anderson et al. (2018). USA	Quantitative; longitudinal/follow-up	IVF	193 heterosexual coupled parents	IVF twins presented fewer externalizing behaviours than IVF singletons in middle childhood
36	Carone et al. (2018). Italy	Mixed methods (qualitative, quantitative, and observational); cross-sectional	surrogacy, sperm donation and egg donation	80 families: 40 gay surrogacy and egg donation coupled father families; 40 lesbian DI coupled mother families	ART Children with gay and lesbian parents presented no significant differences in internalizing and externalizing problems Children with gay fathers had significantly fewer internalizing problems than the normative sample
37	Green et al. (2019). USA	Quantitative and cross-sectional	Surrogacy	68 gay coupled fathers	Daughters of gay fathers presented markedly lower internalizing problems compared with a matched sample of girls from the general population
38	Carone et al. (2020). Italy	Mixed methods (qualitative, quantitative and observational); cross-sectional	Surrogacy and IVF	155 families: 35 gay surrogacy single fathers, 30 heterosexual surrogacy single fathers, 45 gay surrogacy coupled fathers and 45 heterosexual IVF coupled parents	Children from gay/heterosexual single families and, gay/heterosexual partnered families presented no significant differences in internalizing and externalizing problems
39	Chen et al. (2020). USA	Mixed methods (quantitative and observational); cross-sectional	DI, egg donation and other not specified	55 families: 51 heterosexual coupled parents and 4 lesbian coupled mothers	For families with high-conversation orientation, children who knew their conception presented a better psychological adjustment than children who did not know this information
40	(f) Golombok et al. (2020). UK	Mixed methods (qualitative, quantitative, and observational); longitudinal/ follow-up	Sperm donation	81 families: 44 heterosexual single mothers and 37 heterosexual coupled parents	Children from solo mothers and partnered mothers presented no significant differences regarding emotional and behavioural problems
41	Imrie et al. (2020). UK and USA	Mixed methods (qualitative and quantitative); cross-sectional	Not specified	35 families: 22 transgender single parents, 13 transgender and cisgender coupled parents	Children from trans and cisgender families presented good quality relationships with their parents and good psychological adjustment
42	Carone et al. (2021). Italy	Mixed methods (qualitative and quantitative); cross-sectional	Surrogacy and IVF	120 families: 31 gay surrogacy single fathers, 28 heterosexual surrogacy single fathers, 31 gay surrogacy coupled fathers and 30 heterosexual IVF coupled parents	Children from gay (single and partnered) and heterosexual (single and partnered) families presented no differences in any behavioural outcome, showing low levels of internalizing and externalizing problems
43	Díez et al. (2021). Spain	Quantitative; cross-sectional	Not specified	98 families: 45 single mothers (29 adoptive and 16 ART families) and 53 coupled mothers (27 adoptive and 26 ART families)	ART children from single mother families presented a good psychological adjustment and with no significant differences when compared with ART children from two-parent families
44	Carone et al. (2021). USA	Quantitative; longitudinal/ follow-up	DI	74 lesbian families: 26 unknown donor, 26 known donor and 22 open-identity donor;	Children from lesbian families with anonymous, known, or open-identity sperm donor, presented no significant differences in internalizing and externalizing problem behaviours
45	Gervoise-Boyer et al. (2021). France	Quantitative; longitudinal/ follow-up	IVF, ICSI	Families with ART singleton or twins; 295 children	Children from different ART does not appear to present differences in their behaviour and well-being outcomes

a, b, c, d, e and f—same samples

*IVF* in vitro fertilization, *DI* donor insemination, *NC* naturally conceived, *ICSI* intracytoplasmic sperm injection, *OI* oocyte injection, *IUI* intrauterine insemination

Publications started in 1996 with an average of at least one publication per year until 2021, although four particular years summed 16 publications (2007, 2018, 2020 and 2021). The studies’ samples were recruited in eight different countries, with the overwhelming majority of these conducted in the UK (*n* = 16) and/or the USA (*n* = 15), followed by The Netherlands (*n* = 11), Italy (*n* = 6), Spain (*n* = 3), France (*n* = 2), Israel (*n* = 1) and Taiwan (*n* = 1). Studies’ sample sizes ranged from 30 to 769 and the average sample size was 153. Most participants were recruited through either probabilistic national samples or through non-probabilistic sampling (e.g., fertility clinics, hospitals, advertisements in targeted publications, public events, through specialists (snowball) and interest groups). The majority of the studies were cross-sectional (*n* = 26) and 19 studies used prospective longitudinal and/or follow-up research designs. Since some studies are part of the same prospective longitudinal and/or follow-up research, 38 different samples from a total of 45 studies were included in this review. Nineteen studies used a quantitative approach, and 26 studies used a mixed-methods approach (17 quantitative and qualitative studies, 8 quantitative, qualitative and observational studies and 1 quantitative and observational study). Most of the studies used a multi-informant approach (*n* = 27) including parents, children and teachers (*n* = 11 studies), parents and children (*n* = 11 studies), or parents and teachers (5 studies). The remaining studies were conducted solely with parents (17 studies), and 1 study was conducted solely with children.

P*sychological adjustment* was differently conceptualized in the 45 papers, namely, as *socioemotional* development/functioning, *social* development/adjustment/competence, *emotional* development/adjustment/problems/difficulties, *behavioural* adjustment/problems, *psychological* problems/adjustment/well-being. To assess children’s psychological adjustment, the most widely used instruments were the Achenbach System of Empirically Based Assessment (ASEBA) forms [CBCL/6–18, TRF] (18 studies) and the Strengths and Difficulties Questionnaire [SDQ] (22 studies) within a multi-informant approach. Only two studies used the ASEBA and SDQ children’s self-report forms. Most of these studies used the ASEBA’s Total Problem, Internalizing and Externalizing Scales or the SDQ’s Total Difficulties Scales. Only one study [29] used the Internalizing and Externalizing Scales. Almost half of these studies compared their samples’ scores with normative data, whereas the remaining studies compared their samples’ scores with different comparison groups (naturally conceived children or adoptive children). Very few studies (*n* = 6) used the Rutter scales, the Pictorial Scale of Perceived Competence, the Eyberg Child Behaviour Inventory (ECBI), the Rosenberg Self-esteem Scale, and the Social Skills Rating System (SSRS) to examine children’s psychological adjustment.

### Psychological adjustment of children born through ART

#### ART children and normative data

The first specific objective of this review was to assess ART children’s psychological adjustment from different cultural contexts/countries and rated by multi-informants according to the normative data for the standardized indexes of mental health. As such, the mean scores of the Strengths and Difficulties Questionnaire (SDQ [30];) and ASEBA forms (CBCL and TRF [31];) of the children of the included studies were used to perform a comparative analysis with the standardized normative data for those measures (see Appendix B available in the OSF, at https://osf.io/pjnbk/?view_only=92979f986dfc419297eed6a8847cda1e). For the SDQ data interpretation, we followed the original three-band categorization (normal, borderline or abnormal), and for the ASEBA forms the categorization was normal range, borderline clinical range and clinical range. By conducting this analysis, we not only deepen the information that can be drawn from these studies, but also reduce the risk of bias associated with sampling processes, samples’ characteristics and the heterogeneity of the studies’ methodologies. We found that based on both parents’ and teachers’ ratings, children’s psychological adjustment was within the normal range in all the included studies for the SDQ, total scale scores and internalizing/externalizing scores, and for the ASEBA, total scores and internalizing/externalizing scores were also within the normal range.

#### ART children and different ART

Several ARTs were included in the reviewed studies, namely, donor insemination (DI) (*n* = 21), in vitro fertilization (IVF) (*n* = 17), intracytoplasmic sperm injection (ICSI) (*n* = 4), intrauterine insemination (IUI) (*n* = 2), gametes donation [oocyte (*n* = 1) and spermatozoids (*n* = 3)], egg donation (*n* = 7), embryo donation (*n* = 3) and surrogacy (*n* = 11). The overwhelming majority of the studies included DI and/or IVF, although some studies (*n* = 9) have not specified the ART used by the participants.

Given the lack of overall psychological adjustment problems in ART children, we will next examine possible differences according to different ART used, especially ART involving reproductive donation where the child is not genetically related to at least one parent. ART that involve IVF and ICSI could include genetic material from both parents, only one or none. However, for most of the articles with these techniques, it was not possible to discriminate if those families included gamete donation or not. This information should be considered in the following results description.

The vast majority of the studies included DI families in their samples and reported that children were well adjusted. These studies found no significant differences between NC and adoptive children on any of the measures of psychological adjustment [SN1; SN4; SN6; SN9; SN13; SN15; SN21; SN22; SN26]. Nevertheless, for two studies conducted in the 1990s, DI children from heterosexual-parented families presented higher levels of emotional problems than NC, IVF and DI children from lesbian-parented families [SN2; SN3]. From a cross-cultural perspective, Dutch DI children reported higher levels of emotional and behavioural problems than children from the UK, Italy and Spain [SN1], whereas in another study, Dutch DI children presented lower levels of emotional and behavioural problems comparatively to the American children [SN18].

Children born through egg and/or embryo donation also presented good social and emotional development overall [SN6; SN13; SN21; SN22; SN23; SN26; SN36; SN39; SN45] and fewer behavioural (conduct) problems than adoptive children [SN14]. Three studies reported no differences between surrogacy children and NC children during their early school years [SN13; SN21; SN23], although one study reported higher levels of adjustment difficulties in 7-year-old surrogacy children, compared to egg donation, DI and NC children [SN26].

The studies revealed that IVF children presented normal psychological adjustment [SN1; SN3; SN5; SN6; SN7; SN8; SN9; SN10; SN14; SN17; SN21; SN27; SN29; SN35; SN38; SN42; SN45], with fewer behaviour problems in one study [SN7], compared to NC children. The large majority of the studies did not discriminate between children born through IVF with gametes from both parents (homologous IVF) and children born through IVF with gametes or embryo donation. Nevertheless, four studies included children born through homologous IVF [SN7; SN8; SN14; SN42;] and one discriminated the different types of IVF and concluded that children’s psychological adjustment was similar regardless of their genetic or gestational link to their parents, nor do they appear to be at higher risk of psychological adjustment problems in middle childhood [SN21].

#### ART children and different family configurations

The third specific objective was to examine possible differences in children’s psychological adjustment across different family configurations. The majority of the children from single mother or single father families presented good psychological adjustment and high social competences, with no reported significant differences compared to ART children from two-parent families [SN4; SN32; SN38; SN40; SN41; SN42; SN43]. A study in Taiwan that only included single mothers of IVF children reported that they presented fewer behavioural problems than NC children. In one study, children from single mother families presented higher levels of externalizing problems then children from two-parent families [SN24].

Concerning ART children’s psychological adjustment in lesbian- and gay-parented families, the overwhelming majority of the studies found no significant differences between these children and children from heterosexual-parented families in emotional and behavioural difficulties [SN2; SN4; SN16; SN28; SN31; SN33; SN38; SN42]. Further, children from lesbian-/gay-parented families showed no differences in the prevalence of internalizing or externalizing problems when compared to NC children and/or adoptive children [SN11; SN16; SN28; SN30; SN31; SN42; SN44] or normative samples [SN2; SN4; SN20; SN33; SN38]. In fact, some studies revealed that children’s internalizing and externalizing problems from gay-/lesbian-parented families were very low when compared to the cutoff point for clinical problems [SN34; SN36; SN37]. Noteworthy, in two studies, surrogacy children with gay fathers compared to normative samples presented the lowest internalizing problems [SN36], especially the daughters [SN37]. This seems to indicate that there could exist unique family processes in gay father’s surrogacy families.

Two studies concerning ART children from lesbian mothers assessed the influence of the donor status (anonymous or known) in children’s psychological adjustment and found no significance between the two groups [SN15; SN44]. In two other studies, ART children from lesbian/gay single parents presented high levels of self-worth and low levels of internalizing and externalizing problems, and showed no significant differences when compared to ART children from lesbian/gay two-parent families, and heterosexual single- or two-parent families [SN32; SN42]. Nevertheless, in one study with lesbian- and heterosexual-parented families, children from single mother families reported higher levels of externalizing problems when compared to children from two-parent families, regardless of mother’s sexual orientation [SN24].

Only one study with ART children with transgender parents was found and included in this review. The authors used the SDQ to evaluate children’s psychological adjustment and reported that children with transgender parents scored within the normal range for the total difficulties scale. Further, children from both transgender and cisgender-parented families presented high-quality relationships with their parents and good psychological adjustment. Child age at which the transgender parent disclosed their gender identity to their child was not associated with their child’s psychological outcomes [SN41].

## Discussion

The present systematic review aimed to assess the psychological adjustment of ART children aged between 3 and 11 years. The specific objectives were the following: (a) to compare ART children’s scores on standardized indexes of mental health with normative data; (b) to examine possible differences on children’s psychological adjustment across different ART especially those involving reproductive donation; (c) and to examine possible differences in children’s psychological adjustment across different family configurations, namely between children from single mother/father families versus coupled parents’ families, lesbian-parented families and/or gay-parented families versus heterosexual-parented families and transgender-parented families versus cisgender-parented families.

Overall, there were no substantial differences between ART children and NC or adoptive children, or differences for internalizing and externalizing problems when ART children were compared with children from normative samples. Almost all studies included in this review have used the Strengths and Difficulties Questionnaire (SDQ) or the Achenbach System of Empirically Based Assessment (ASEBA) within a multi-informant approach which enables the assessment of children’s psychological adjustment through the standardized indexes provided by these worldwide recognized instruments. We confirmed that similarly to natural conceived children, all ART children examined with the aforementioned measures were under the clinical range for psychiatric disorders.

Further, most of the studies have also used a multi-methods approach which helped to complement the information provided by the questionnaires, since they are limited to capture the respondents’ perspectives on particular questions that are presented [32]. Looking specifically at the SDQ, only one study [33] used the three subscales versions (Internalizing, Externalizing and Prosocial Behaviours), whereas the other studies used the five subscales version (Peer Problems, Emotional Problems, Behaviour Problems, Conduct Problems and Prosocial Behaviours). This particular aspect of the SDQ should be taken into future consideration, since the three versions could be a more suitable and accurate approach to compare multi-informants across the non-clinical population, as mentioned by the scale creators [30] and other authors (e.g., Costa et al. [34]).

Another goal was to examine possible differences on children’s psychological adjustment according to different ART, mainly the techniques that involve gametes and/or egg donation. For most of the studies, no statistically significant differences were found for main indexes of emotional and behavioural problems. Nevertheless, two studies [35, 36] conducted in the 1990s reported some differences between DI children and other ART children and concluded that the differences lean on the DI heterosexual-parented families who reported higher adjustment difficulties compared to DI children from lesbian-parented families and other comparison groups. In these studies, DI heterosexual families were more secretive than IVF families and lesbian-parented families. One possible explanation is the possible influence of the confidentiality and the taboo surrounding male infertility on children’ emotional and behavioural adjustment. Nonetheless, this hypothesis was not verified in other studies included in this review. Another study that did not corroborate the absence of differences between children from different ARTs was a study performed with heterosexual-parented families that have pursued surrogacy and reported higher psychological adjustment problems in surrogacy children when compared to other ART techniques at the age of 7 years [37]. The authors have raised the hypothesis that the absence of a gestational connection between parents and their child could be more problematic for children than the absence of a genetic relationship; however, this difference was not verified when the children were 10 years old. Also, this difference regarding the adjustment was not indicative of psychological disorder, since the surrogacy children SDQ scores were within the normal range. Possibly, this could be related to the time that children begun to ask questions about their origins, since by the age of 6–8 years children start understanding the significance of the biological concept of family and the repercussions of a non-biological connection between family members [38].

Differently from the results, other studies with lesbian and gay-parented families concluded that children from surrogacy gay fathers not only were well adjusted as other ART children or comparison groups, as they were the group with the lowest psychological adjustment problems when compared with normative children [33, 39, 41]. For one particular study, it was the girls who presented the lowest levels of adjustment problems [41]. Similar results were observed in children from families with adoptive gay fathers. These children presented less internalizing/externalizing behaviours, and less gender-stereotyping comparatively with children from heterosexual adoptive families [41, 42]. A recent meta-analysis that included studies mostly with surrogacy gay also found that children of gay fathers may present less internalizing and externalizing problems than children of heterosexual parents and highlighted that these differences could be assigned to parents’ specific sociodemographic characteristic such as higher income and higher education level, and also to an increased resilience and with more egalitarian parenting roles [23]. The degendered parenting associated with some gay fathers, as mentioned in Carneiro and colleagues’ (2017) systematic review, could also be promote these findings [22]. It seems important that future studies rather than just focusing on the different ART and their impact on children’s psychological adjustment could expand the evaluation and assess how the families manage the disclosure and the absence of genetic and/or gestational link with their children. The mentioned differences should be further analysed and contextualized with other family specificities (e.g., family communication, parental style, quality of parental–children relationship).

Other purpose of the present review was to assess whether children from different family configurations present differences on their psychological adjustment. Children from the included studies seem to be well adjusted regardless of their parents’ sexual orientation or number of family members. Heterosexual coupled-parented families return to this technology most likely due to the infertility condition of at least one of the couple members, which is very different from the reason why lesbian, gay and heterosexual single parents pursue this path. In the present study, ART children from lesbian- and gay-parented families do not seem to differ from other comparison groups, nor from children from normative samples for the main psychological adjustment indexes. In fact, for some studies children from lesbian/gay-parented families presented the lowest levels of adjustment problems when compared with children from normative samples [15, 28, 40]. Almost all the studies with single-parent families concluded singlehood does not negatively influence ART children’s psychological adjustment, except for one study with Israeli mothers where children from single-mother families reported more psychological adjustment difficulties than children from coupled mother families regardless of their sexual orientation [43]. Interestingly, a study in Taiwan performed only with single mothers of IVF children concluded that they presented fewer behavioural problems than NC children. The differences mentioned could be because of cultural differences between Western and Eastern countries that should be further investigated.

Although there are several studies assessing the influence of parent’s sexual orientation and ART children’s psychological adjustment, only one study was found with the purpose of evaluating the influence of the gender identity on ART children’s psychological adjustment. This study [39] concluded that children from transgender-parented families were well adjusted with high-quality relationships with their parents. The empirical literature on children’s developmental outcomes on transgender-parented families remains very limited. Due to the increasing awareness of trans equality issues in some countries, these families will become more numerous and noticeable in the coming years [44]; therefore, future studies should conduct more studies with families with trans parents and also adopting sophisticated methodological approaches to understand this new family configuration [1].

There are some limitations arising from this study that should be take into account while interpreting the findings of this review. The retention rates were not included and discriminated in the outcome analysis, nor the levels of agreement of teachers and parents about children’s psychological adjustment. Another limitation of the findings of this review is that we did not include specifically bisexual parents and it is an important and scarce topic in the literature field. Futures studies should focus on these families. As mentioned earlier, studies with transgender-parented families should be conducted to evaluate the children’s well-being and to introduce more information about this family configuration. Also, future studies should address the disclosure issues and understand its possible impact on children’s psychological adjustment. Future studies should also compare the disclosure outcomes according to children’s age to verify if there are significant differences or a more appropriate age to disclose. The impact of the donor status for the family and children’s adjustment should also be further addressed.

In sum, all these different families arise from different contexts and with different restrictions or limitation to their parenthood pathways and probably this could influence children’s psychological adjustment differently but not necessarily negatively. Those idiosyncratic differences will require different psychological resources and strategies to navigate throughout those pathways, which will impact their further decisions to disclose to their children, family members, friends, and acquaintances. The families need to find out what narrative and what strategies would be more suitable for them to maintain or promote good family functioning. This should be taken into account by family practitioners and clinicians to help parents mitigate the impact of these potential stressors and to be more psychologically adjusted to parenthood, and also help them to minimize the impact of these potential stressors, since there are other inevitable stressors inherent to parenthood that are present in all families. The results of this systematic review enable practitioners to develop an informed view of ART children’s psychological adjustment. Also, despite the multiplicity of factors that influence ART families and their children’s psychological health, these results highlight how ART children from different family configurations (heterosexual/lesbian/gay/transexual parents or coupled/single parent(s)) are all similarly psychologically adjusted and without the presence of clinical problems.

## List of the references of the included studies

SN1: Golombok S, Brewaeys A, Cook R et al. (1996) The European study of assisted reproduction families: family functioning and child development. *Hum Reprod* 11(10):2324–2331. 10.1093/oxfordjournals.humrep.a019098
SN2: Brewaeys A, Ponjaert I, Van Hall EV, et al (1997a) Donor insemination: child development and family functioning in lesbian mother families. *Hum Reprod* 12(6):1349-1359. 10.1093/humrep/12.6.1349
SN3: Brewaeys A, Golombok S, Naaktgeboren N, et al (1997b) Donor insemination: Dutch parents’ opinions about confidentiality and donor anonymity and the emotional adjustment of their children. *Hum Reprod* 12(7):1591-1597. 10.1093/humrep/12.7.1591.
SN4: Chan RW, Raboy B, Patterson CJ (1998) Psychosocial adjustment among children conceived via donor insemination by lesbian and heterosexual mothers. *Child Dev* 69(2):443-457. 10.2307/1132177.
SN5: Montgomery TR, Aiello F, Adelman RD, et al (1999) The psychological status at school age of children conceived by in-vitro fertilization. *Hum Reprod* 14(8):2162-2165. 10.1093/humrep/14.8.2162.
SN6: Golombok S, Murray C (1999) Social versus biological parenting: Family functioning and the socioemotional development of children conceived by egg or sperm donation. *J Child Psychol Psychiatry* 40(4):519-527. 10.1111/1469-7610.00470.
SN7: Hahn CS, DiPietro JA (2001) In vitro fertilization and the family: quality of parenting, family functioning, and child psychosocial adjustment. *Dev Psychol* 37(1):37-48.
SN8: Golombok S, MacCallum F, Goodman E (2001) The “test‐tube” generation: Parent–child relationships and the psychological well‐being of in vitro fertilization children at adolescence. *Child Dev* 72(2):599-608. 10.1111/1467-8624.00299
SN9: Golombok S, Brewaeys A, Giavazzi MT, et al (2002) The European study of assisted reproduction families: the transition to adolescence. *Hum Reprod* 17(3):830-840. 10.1093/humrep/17.3.830
SN10: Tully LA, Moffitt TE, Caspi A (2003) Maternal adjustment, parenting and child behaviour in families of school‐aged twins conceived after IVF and ovulation induction. *J Child Psychol Psychiatry* 44(3):316-325. 10.1111/1469-7610.00124.
SN11: Bos HM, Van Balen F, Van Den Boom DC, et al (2004) Minority stress, experience of parenthood and child adjustment in lesbian families. *J Reprod Infant Psychol* 22(4):291-304. 10.1080/02646830412331298350.
SN12: Lycett E, Daniels K, Curson R, et al (2004) Offspring created as a result of donor insemination: a study of family relationships, child adjustment, and disclosure. *Fertil Steril* 82(1):172-179. 10.1016/j.fertnstert.2003.11.039
SN13: Golombok S, Murray C, Jadva V, et al (2006) Non-genetic and non-gestational parenthood: consequences for parent–child relationships and the psychological well-being of mothers, fathers and children at age 3. *Hum Reprod* 21(7):1918-1924. 10.1093/humrep/del039
SN14: MacCallum F, Golombok S, Brinsden P (2007) Parenting and child development in families with a child conceived through embryo donation. *J Fam Psychol* 21(2):278-287. 10.1037/0893-3200.21.2.278
SN15: Bos HM, Hakvoort EM (2007) Child adjustment and parenting in planned lesbian families with known and as-yet unknown donors. *J Psychosom Obstet Gyneacol* 28(2):121-129. 10.1080/01674820701409793.
SN16: Bos HM, Van Balen F, Van Den Boom DC (2007) Child adjustment and parenting in planned lesbian‐parent families. *AM J Orthopsychiatry* 77(1):38-48. 10.1037/0002-9432.77.1.38.
SN17: Golombok S, Olivennes F, Ramogida C, et al (2007) Parenting and the psychological development of a representative sample of triplets conceived by assisted reproduction. *Hum Reprod* 22(11):2896-2902. 10.1093/humrep/dem260.
SN18: Bos HMW, Gartrell NK, van Balen F, et al (2008) Children in planned lesbian families: A cross-cultural comparison between the united states and the netherlands. *Am J Orthopsychiatry* 78(2):211-219. 10.1037/a0012711.
SN19: Bos HMW, Van Balen F (2008) Children in planned lesbian families: Stigmatisation, psychological adjustment and protective factors. *Cult Health Sex* 10(3):221-236. 10.1080/13691050701601702.
SN20: Bos HMW, Gartrell NK, Peyser H, et al (2008) The USA national longitudinal lesbian family study (NLLFS): Homophobia, psychological adjustment, and protective factors. *J Lesbian Stud* 12(4):455-471. 10.1080/10894160802278630.
SN21: Shelton KH, Boivin J, Hay D, et al (2009) Examining differences in psychological adjustment problems among children conceived by assisted reproductive technologies. *Int J Behav Dev* 33(5):385-392. 10.1177/0165025409338444.
SN22: Golombok S, Readings J, Blake L, et al (2011) Children conceived by gamete donation: psychological adjustment and mother–child relationships at age 7. *J Fam Psychol* 25(2):230-9. 10.1037/a0022769.
SN23: Golombok S, Readings J, Blake L, et al (2011) Families created through surrogacy: Mother-child relationships and children’s psychological adjustment at age 7. *Dev Psychol* 47(6):1579–1588. 10.1037/a0025292.
SN24: Shechner T, Slone M, Lobel TE, et al (2013) Children's adjustment in non‐traditional families in Israel: The effect of parental sexual orientation and the number of parents on children's development. *Child Care Health Dev* 39(2):178-184. 10.1111/j.1365-2214.2011.01337.x.
SN25: Freeman T, Golombok S (2012) Donor insemination: a follow-up study of disclosure decisions, family relationships and child adjustment at adolescence. *Reprod BioMed Online* 25(2):193-203. 10.1016/j.rbmo.2012.03.009.
SN26: Golombok S, Blake L, Casey P, et al (2013) Children born through reproductive donation: a longitudinal study of psychological adjustment. *J Child Psychol Psychiatry* 54(6):653-660. 10.1111/jcpp.12015.
SN27: Anderson KN, Koh BD, Connor JJ, et al (2014) Twins conceived using assisted reproduction: parent mental health, family relationships and child adjustment at middle childhood. *Hum Reprod* 29(10):2247-2255. 10.1093/humrep/deu190.
SN28: Baiocco R, Santamaria F, Ioverno S, et al (2015) Lesbian mother families and gay father families in Italy: Family functioning, dyadic satisfaction, and child well-being. *Sex Res Social Policy* 12(3):202-212. 10.1007/s13178-015-0185-x.
SN29: Anderson KN, Rueter MA, Connor JJ, et al (2015) Conformity expectations: Differential effects on IVF twins and singletons’ parent–child relationships and adjustment. *J Fam Psychol* 29(4):558-567. 10.1037/fam0000122.
SN30: Tornello SL, Sonnenberg BN, Patterson CJ (2015) Division of labor among gay fathers: Associations with parent, couple, and child adjustment. *Psychol Sex Orientat Gend Divers* 2(4):365-375. 10.1037/SGD0000109.
SN31: Bos HMW, Knox J, van Rijn-van Gelderen L, et al (2016) Same-sex and different-sex parent households and child health outcomes: Findings from the National Survey of Children’s Health. *J Dev Behav Pediatr* 37(3):179-87. 10.1097/DBP.0000000000000288.
SN32: Golombok S, Zadeh S, Imrie S, et al (2016) Single mothers by choice: Mother–child relationships and children’s psychological adjustment. *J Fam Psychol* 30(4):409-418. 10.1037/fam0000188.
SN33: Bos HMW, Kuyper L, Gartrell NK (2018) A population‐based comparison of female and male same‐sex parent and different‐sex parent households. *Fam Process* 57(1):148-164. 10.1111/famp.12278.
SN34: Golombok S, Blake L, Slutsky J, et al (2018) Parenting and the adjustment of children born to gay fathers through surrogacy. *Child Dev* 89(4):1223-1233. 10.1111/cdev.12728.
SN35: Anderson KN, Rueter MA, Connor JJ, et al (2019) Parental conformity expectations' effect on twins' and singletons' parent–adolescent relationships: Associations with change in adjustment from middle childhood to adolescence. *J Res Adolesc* 29(4):832-845. 10.1111/jora.12416.
SN36: Carone N, Lingiardi V, Chirumbolo A, et al (2018) Italian gay father families formed by surrogacy: Parenting, stigmatization, and children’s psychological adjustment. *Dev Psychol* 54(10):1904-1916. 10.1037/dev0000571.
SN37: Green RJ, Rubio RJ, Rothblum ED, et al (2019) Gay fathers by surrogacy: prejudice, parenting, and well-being of female and male children. *Psychol Sex Orientat Gend Divers* 6(3):269-283. 10.1037/sgd0000325.
SN38: Carone N, Baiocco R, Lingiardi V, et al (2020) Gay and heterosexual single father families created by surrogacy: Father–child relationships, parenting quality, and children’s psychological adjustment. *Sex Res Social Policy* 17(4):711-728. 10.1007/s13178-019-00428-7.
SN39: Chen M, Rueter MA, Anderson KN, et al (2020) Conversation orientation moderates the relationship between information sharing of medically assisted reproduction and child adjustment. *Fam Process* 59(1):229-243. 10.1111/famp.12415.
SN40: Golombok S, Zadeh S, Freeman T, et al (2020) Single mothers by choice: Parenting and child adjustment in middle childhood. *J Fam Psychol* 35(2):192-202. 10.1037/fam0000797.
SN41: Imrie S, Golombok S (2020) Impact of new family forms on parenting and child development. *Annu Rev Dev Psychol* 2:295–316. 10.1146/annurev-devpsych-070220-122704.
SN42: Carone N, Barone L, Lingiardi V, et al (2021) Factors associated with behavioral adjustment among school-age children of gay and heterosexual single fathers through surrogacy. *Dev Psychol* 57(4):535-547. 10.1037/dev0001155.
SN43: Díez M, González M, Morgado B (2021) Single mothers by choice in Spain: Parenting and psychosocial adjustment in adopted and ART children. *J Fam Psychol* 35(6):767–779. 10.1037/fam0000680.
SN44: Carone N, Gartrell NK, Rothblum ED, et al (2021) The stability of psychological adjustment among donor-conceived offspring in the US National Longitudinal Lesbian Family Study from childhood to adulthood: Differences by donor type. *Fertil Steril* 115(5):1302-1311. 10.1016/j.fertnstert.2020.12.012.
SN45: Gervoise-Boyer MJ, Anzola AB, Sambuc R, et al (2021) Health and well-being outcomes of adolescents conceived through in vitro fertilization and intracytoplasmic sperm injection. *Reprod Sci* 28(5):1428-1438. 10.1007/s43032-020-00407-z.
